# Polymerization-Driven Immobilization of dc-APGD Synthesized Gold Nanoparticles into a Quaternary Ammonium-Based Hydrogel Resulting in a Polymeric Nanocomposite with Heat-Transfer Applications

**DOI:** 10.3390/polym10040377

**Published:** 2018-03-29

**Authors:** Piotr Cyganowski, Anna Dzimitrowicz, Piotr Jamroz, Dorota Jermakowicz-Bartkowiak, Pawel Pohl

**Affiliations:** 1 Department of Polymer and Carbonaceous Materials, Faculty of Chemistry, Wroclaw University of Science and Technology, 27 Wybrzeze St. Wyspianskiego Street, 50-370 Wroclaw, Poland; piotr.cyganowski@pwr.edu.pl (P.C.); dorota.jermakowicz-bartkowiak@pwr.edu.pl (D.J-B.); 2 Department of Analytical Chemistry and Chemical Metallurgy, Faculty of Chemistry, Wroclaw University of Science and Technology, 27 Wybrzeze St. Wyspianskiego Street, 50-370 Wroclaw, Poland; piotr.jamroz@pwr.edu.pl (P.J.); pawel.pohl@pwr.edu.pl (P.P.)

**Keywords:** atmospheric pressure plasma, metallic nanostructures, nanocomposite, resin

## Abstract

A new method for the production of nanocomposites, composed of gold nanoparticles (AuNPs) and (vinylbenzyl)trimethylammonium chloride-*co*-*N*,*N*-methylene bisacrylamide (VBTAC-*co*-MBA) hydrogel, is described. Raw-AuNPs of defined optical and granulometric properties were synthesized using direct current atmospheric pressure glow discharge (dc-APGD) generated in contact with a solution of HAuCl_4_. Different approaches to the polymerization-driven synthesis of Au/VBTAC-*co*-MBA nanocomposites were tested. It was established that homogenous dispersion of AuNPs in this new nanomaterial with was achieved in the presence of NaOH in the reaction mixture. The new nanocomposite was found to have excellent heat-transfer properties.

## 1. Introduction

Nanotechnology has gained a great deal of attention in societies all over the world. The production and utilization of various nanomaterials (NMs) is recognized as a major factor facilitating an improved quality of human life. This includes enhancement of human health [1,2], conservation of cultural heritage [3], production or management of energy resources [4], and so on. In particular, gold nanoparticles (AuNPs) are especially important due to their medical applications in drug delivery systems, catalysis, biosensoring [5], and fuel-cell technology [6].

The usage of NPs in real-world applications has serious obstacles. Prime examples include difficulties in synthesizing AuNPs of specific size and shape, as well as in purification of Au nanostructures from post-reaction mixtures that also contain unreacted reagents and possibly toxic byproducts [7]. A potential solution to this problem is to synthesize stable-in-time Au nanostructures through the use of atmospheric pressure plasma (APP)-based methods [8,9,10,11,12,13,14,15,16,17]. In APP-based methods, APP itself serves as a source of reducing potential, negating the requirement for the inclusion of additional, potentially toxic reducing agents [8,9,10,11,12,13,14,15,16,17]. The reduction of metal ions to their metallic, nanometric forms is mediated through the production of reactive oxygen and nitrogen species (RONS) at the APP-liquid interface [15,16,17]. The simple and low-cost process of using APP-based methods, together with the lack of a requirement for additional reducing agents, has led to these methods attracting significant scientific interest.

Most APP-based reaction-discharge systems used for synthesis of AuNPs work in a stationary (i.e., non-flowing) mode. This can impair the ability of APP-based reduction methods to be used in the production of size- and shape-defined AuNPs as it is not easy to control plasma-liquid interactions. The non-flowing nature of this process limits the rate that Au nanostructures may be obtained. For these reasons, our research group has developed a reaction-discharge system that functions in a continuous-flow mode [15,16]. In this system, APP, i.e., direct current atmospheric pressure glow discharge (dc-APGD), is operated between the surface of a flowing liquid electrode and a gaseous nozzle jet [15,16]. To reduce the cost of AuNPs synthesis, the gaseous nozzle jet can be sustained by a solid metallic electrode [17]. In both systems, it is possible to produce size- and shape-controlled Au nanostructures by changing the precursor concentration, the discharge current value, and the flow rate of the flowing liquid solution acting as electrode [16,17]. Moreover, under defined reaction-discharge conditions of developed dc-APGD-based systems, there is no need to add additional stabilizers due to electrostatic stabilization of AuNPs caused by the accumulation of negative charges on their surfaces [9,17]. 

One intriguing use of AuNPs is for heat-transfer applications. The term heat transfer refers to the thermal management of advanced appliances including miniaturized electronics, optical devices, and systems for energy generation and storage. Efficient and programmable heat transfer addresses major societal concerns such as energy-efficiency and energy saving [18,19,20]. Several types of NPs have been investigated for their potential use in improving heat transfer. This includes suspensions of NMs such as ceramics, carbon nanotubes [18,21], and different types of metallic [20] and non-metallic NP suspensions [21], often called nanofluids (NFs). Within all these cases, suspensions of NMs are reported to reveal an extraordinary ability to increase heat exchange, enabling, for example, higher heat conduction and microchannel cooling [18,20]. Among them, NFs containing AuNPs are particularly interesting, as they are effective even at ultra-low (0.001 vol %) concentrations [22,23]. However, despite the advantages of such solutions, obstacles to their use include reduced stability of NPs and the difficulty of using solvent-NP suspensions in devices powered by an electric current [18,20,21]. Overcoming of these challenges can be achieved by the immobilization of NMs in polymeric matrices [19] creating hydrogel-based nanocomposites [24].

Polymeric nanocomposites are a unique type of NMs that can consist of metallic NPs dispersed in a polymeric phase. These materials can be used as biosensors [25], optical devices [26], or a platform for the immobilization of proteins [27]. The utility of nanocomposites is associated with the type of the polymeric matrix as well as the nanostructures dispersed within it. The stability of such materials depends on the proper immobilization of the NPs in the polymer. Hence, successful immobilization of AuNPs within a polymeric network can be achieved by incorporating appropriate functional groups into the latter. For example, previous works demonstrated that the presence of quaternary ammonium groups in a polymer improves the stabilizing abilities of nanocomposites [28]. The application of amino-ligands that have an affinity towards Au species [29,30,31,32] also cannot be excluded.

Generally, two approaches are applied for the synthesis of polymer-based nanocomposites: (1) synthesis of nanostructures during reduction-coupled sorption of a precursor onto functional groups of a polymer (in situ); or (2) production of NPs outside the polymeric network followed by their immobilization (ex situ) [32,33]. In situ synthesis is carried out as a single step, omitting application of additional reducing agents and immobilization of NPs [32,34]. However, it is very difficult to control the properties of the resultant nanostructures, as the final outcome of synthesis relies on the nature of functional groups acting as nanoreactors. A more convenient approach is ex situ production, which allows immobilization of specific NPs whose characteristics are easier to control. Reduced precursors of noble metals have been successfully immobilized within a variety of polymers [7,28,35,36,37] as well as biopolymers [7,38,39]. Among them, the polymeric resins bearing strong base amino functionalities play the most significant role, as the quaternary ammonium groups reveal strong affinity towards ionic forms of noble metals and a proven potential for the immobilization of Au nanostructures [28]. The main drawback suffered by the ex situ approach is related to diffusion limitations, as the migration of nanostructures within a cross-linked polymeric network is seriously limited. As such, the rate of immobilization is often poor. Within the present study, we propose a solution for this issue by introducing NPs to a mixture of water-soluble monomers, which is then subjected to polymerization. Such a polymerization-driven approach guarantees proper dispersion of metallic structures within the liquid phase, which are then immobilized during the polymerization process. 

There are plenty of polymers that can be applied for polymerization-driven immobilization of AuNPs [40]; however, only those compatible with the water-based medium used in the reaction-discharge system can be applied. This seriously limits available choices for the polymeric matrix, which must contain hydrophilicity (in case of ex situ synthesis followed by immobilization) or, as in the present study, the monomers that the matrix is made from must be soluble in water (polymerization-driven immobilization). The selection of quaternary ammonium-based hydrogels will fulfill all of these requirements, as they are water-soluble monomers with strong-base amino functionality and chemical stability (due to possible cross-linking). Moreover, quaternary ammonium-based hydrogels have reversible hydrophilic-hydrophobic properties, which significantly broaden their possible applications for biomedical purposes [41] and separation processes [42]. Additionally, they are proven to be suitable for the synthesis of nanocomposites [43].

For this reason, the aim of the present work was to produce a Au/VBTAC-*co*-MBA nanocomposite, consisting of AuNPs immobilized within a (vinylbenzyl)trimethylammonium chloride-*co*-*N*,*N*-methylene bisacrylamide (VBTAC-*co*-MBA) hydrogel. To the best of our knowledge, this is the first work in which a solution containing raw-AuNPs generated by dc-APGD was immediately used as a reaction environment for VBTAC and MBA monomers. Within this research, different approaches for polymerization-driven synthesis were assessed to determine the conditions under which a Au/VBTAC-*co*-MBA nanocomposite with homogenous dispersion and defined optical and granulometric properties of immobilized AuNPs was obtained. The designed product was then investigated toward its possible application in heat-transfer processes. Its ability to release temperature in air-cooling systems simulating modern electronic devices was monitored.

## 2. Materials and Methods

### 2.1. Reagents and Solutions

All reagents were of analytical quality or better. Re-distilled water was used in all experiments. Chloroauric acid tetrahydrate (HAuCl_4_ × 4H_2_O) was obtained from Avantor Performance Materials (Gliwice, Poland) and used for the preparation of a stock solution with a Au(III) ions at a concentration of 750 mg·L^−1^. Afterwards, the prepared stock solution was diluted 10-fold to obtain a working solution with a final Au(III) concentration of 75 mg·L^−1^. VBTAC, MBA, as well as potassium persulfate (K_2_S_2_O_8_, >99.9%) were purchased from Sigma-Aldrich (Poznan, Poland) and used in the polymerization procedure.

### 2.2. Polymerization-Driven Immobilization of Raw Gold Nanoparticles (AuNPs) in Quaternary Ammonium-Based Hydrogel

**Figure 1:**
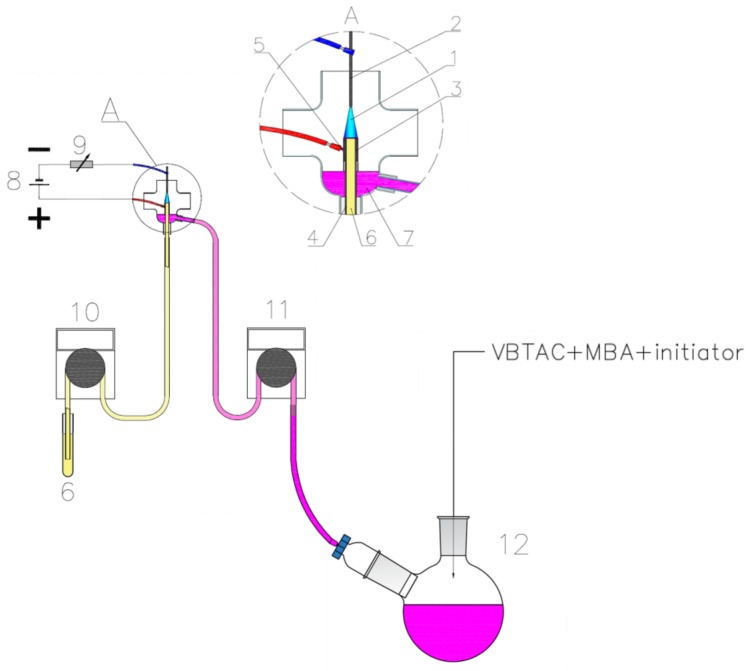
Developed system for the production of Au/VBTAC-*co*-MBA nanocomposite. 1—direct current atmospheric pressure glow discharge (dc-APGD), 2—a tungsten cathode, 3—a graphite tube, 4—a quartz capillary, 5—a platinum wire attached to the graphite tube, 6—a gold nanoparticles (AuNPs) precursor solution, 7—raw-AuNPs, 8—the dc-HV supply, 9—ballast resistor, 10—first peristaltic pump, 11—second peristaltic pump, 12—a round-bottom flask placed onto a magnetic stirrer equipped with a heating plate.

Synthesis of nanocomposites was carried out using an apparatus composed of (1) a reaction discharge system for the production of raw-AuNPs, which were then directly introduced to (2) a vessel containing monomers and the initiator, as displayed in Figure 1.

Raw-AuNPs were synthesized by applying the method described by Dzimitrowicz et al. (2016) [17]. Briefly, production of Au nanostructures was carried out in a continuous-flow reaction-discharge system, in which dc-APGD was operated in a 5.0 mm gap between the surface of a flowing liquid anode (FLA) and the sharpened tip of a solid tungsten cathode (ID = 4.0 mm, Figure 1). The FLA solution was introduced to the developed reaction-discharge system through a quartz capillary (OD = 4.0 mm; ID = 2.0 mm), onto which a graphite tube was mounted (OD = 6.0 mm; ID = 4.0 mm), by a four-channel peristaltic pump (MasterFlex L/S, Cole-Parme^®^, Vernon Hill, IL, USA), at a flow rate of 3.0 mL·min^−1^. To charge FLA solution, a platinum wire was connected to the quartz-graphite capillary. A dc-HV potential of 1300 V was supplied to both electrodes from a dc-HV supply (Dora Electronics Equipment, Wroclaw, Poland). The discharge current was set to 50 mA using a 10 kΩ ballast resistor (Tyco Electronics, Berwyn, IL, USA). The entire reaction-discharge system was located inside a semi-closed quartz chamber. 

The solution containing the synthesized raw-AuNPs with unreacted precursor was then directly transferred using a second peristaltic pump (MasterFlex L/S, Cole-Parme^®^, Vernon Hill, IL, USA), at a flow rate of 3.0 mL·min^−1^, to a round-bottom flask containing VBTAC, MBA (15 mol % in respect to VBTAC), and potassium persulfate (5 mol % in respect to monomers). The amount of the solution introduced was kept to a minimum (approximately 8 mL), allowing solid reagents to be dissolved. When reagents were completely dissolved, the resultant mixture was degassed with N_2_ and temperature was elevated to 75 °C using an IKA RCT Basic magnetic stirrer (Staufen im Breisgau, Germany) equipped with a round heating dish and a reflux condenser to carry out free-radical polymerization according to the procedure described by Cyganowski et al. [42]. As the solution after dc-APGD treatment contained raw-AuNPs as well as their unreacted precursor, three different approaches were utilized for polymerization-driven immobilization of synthesized Au nanostructures according to the following procedures:

(i). NC1: Free-radical polymerization was carried out at 75 °C for 24 h. The resultant product was extensively washed with re-distilled water on a frittered glass funnel until the pH of the outflow was neutral. It was then dried and used for further procedures.
(ii). NC2: The process was performed as was described in the procedure (i) with the difference that the polymerization was run until the gelation point (up to 30 min). Next, the reaction mixture was quickly cooled down and the resultant product was washed with re-distilled water in order to remove excessive AuNPs precursor.
(iii). NC3: The process was carried out as in the procedure (i) with the difference that the reaction mixture was prepared with the addition of NaOH at a 2:1 molar ratio with respect to VBTAC.

The obtained samples were named as NC1, NC2, and NC3 according to synthesis routes described above, respectively. Additionally, raw VBTAC-*co*-MBA copolymer was synthesized [42] and used as a control sample.

### 2.3. Characterization of the Obtained Polymeric Nanocomposites 

In order to assess the optical properties of the raw-AuNPs, ultraviolet-visible (UV–Vis) absorption spectrophotometry was used. A Specord 210 spectrophotometer (Analytic Jena, Jena, Germany) was applied for that purpose. The UV–Vis absorption spectrum of a dc-APGD treated solution containing AuNPs was recorded after 10 min from the dc-APGD treatment in the range from 300 to 800 nm. The re-distilled water was used as a reference sample.

The size distribution by number of the raw-AuNPs as well as their polydisperisty index (PDI) was determined using dynamic light scattering (DLS) by a Photocor Complex device (Photocor Instruments, Tallin, Estonia). The apparatus was equipped with a 657.04 nm/36 mW laser. All measurements were carried out in round vials, submerged in a refractive index-matching liquid (decalin). The scattering angle was set at 90°. Results were analyzed using DynaLS software (Alango Ltd., Tirat Carmel, Israel). All measurements were performed for raw-AuNPs colloidal suspensions at a temperature of 21.96 °C. Corresponding water viscosity was 0.9864 mPa·s^−1^. Size distribution by number and PDI were established as an average value for 3 independent measurements.

Next, the ξ-potential of the raw-AuNPs synthesized *via* dc-APGD was estimated using a Zetasizer Nano-ZS instrument (Malvern Instrument, Worcestershire, UK) with a detector angle of 173°. All measurements were performed in a homogenous square polystyrene cuvette at 25 °C. The presented ξ-potential value was averages of three runs. The Dispersion Technology Software-Zetasizer Software was applied for the data evaluation.

Morphology of the resultant nanocomposites (samples nos. NC1 and NC3) was estimated using a FEI Tecnai G220 X-TWIN (FEI, Hillsboro, OR, USA) transmission electron microscope (TEM) equipped with an energy dispersive X-ray (EDS) module (AZtecEnenrgy, Oxford Instruments, Abingdon, UK). To reach this aim, one drop of given solutions containing nanocomposites was placed onto a Cu grid (CF 400 Cu-UL, GF MICROSYSTEMS, Poznan, Poland) and evaporated to dryness. In order to examine the granulometric properties of Au nanostructures immobilized into quaternary ammonium-based hydrogel (samples nos. NC1 and NC3), the FEI software (version 3.2 SP6 build 421, FEI, USA) was applied. Furthermore, the size distribution of raw-AuNPs in Au/VBTAC-*co*-MBA nanocomposite (sample NC3) was assessed using ImageJ software (version 1.51r, Bethesda, MD, USA) [44], measuring diameters of 100 single NPs. 

Photographs of nanocomposites were obtained in the following way: each of the resultant products after extraction in water was placed on a petri dish and dried at room temperature. Then, a dish with a polymer was placed on white filter paper and photographs were taken against the white background using a Nikon D5200 digital camera (Tokyo, Japan).

To verify the presence of functional groups within the structure of the VBTAC-*co*-MBA copolymer, Fourier transform infrared spectroscopy (FT-IR) was used. The FT-IR spectrum of VBTAC-*co*-MBA was recorded in the range from 4000 to 400 cm^−1^ and 600 to 100 cm^−1^, respectively, by a Vertex 70v FTIR spectrophotometer (Bruker, Bremen, Germany) with a resolution of 4 cm^−1^ and taking 64 scans. The FT-IR measurements were performed under vacuum conditions. 

### 2.4. Assessing the Potential of the Nanocomposite for Heat Transfer 

The synthesized raw VBTAC-*co*-MBA copolymer and Au/VBTAC-*co*-MBA nanocomposite (sample NC3) were tested for their potential use as heat-transfer enhancers. For this purpose, dry copolymer or nanocomposite (0.07 g) were placed in cylindrical glass reaction tubes and then swollen overnight in 20 mL of re-distilled water. Next, the glass tubes with a polymeric materials in water were introduced into an ERTEC Microwave Reactor 02–02 (Wroclaw, Poland) equipped with reflux condenser, magnetic stirrer, and a fast air-cooling system. The copolymer-water and nanocomposite-water mixtures were heated up to 80 °C with microwaves using a power of 200 W for 5 min. Then, the microwave field was turned off and the in-built air-cooling system was engaged. The system, involving a radiator and a fan, allowed for simulation of the transfer of heat in electronic devices. The temperature of the contents of the both glass tubes was monitored until they were cooled to 40 °C. The rate of heat exchange (*k*_he_) within was calculated using Newton’s law of cooling [45] in its simplified form employing the following Equation (1):

(1) $$\frac{dT(t)}{dt}=k_{\mathrm{he}}\cdot \Delta T(t)$$

where *T*(*t*) is temperature in function of time; *k*_he_ is the rate of cooling (s^−1^), and Δ*T*(*t*) is defined as the difference between the temperature over time *t*.

## 3. Results and Discussion

### 3.1. Application of Direct Current Atmospheric Pressure Glow Discharge (dc-APGD) for the Synthesis of Raw-AuNPs

**Figure 2:**
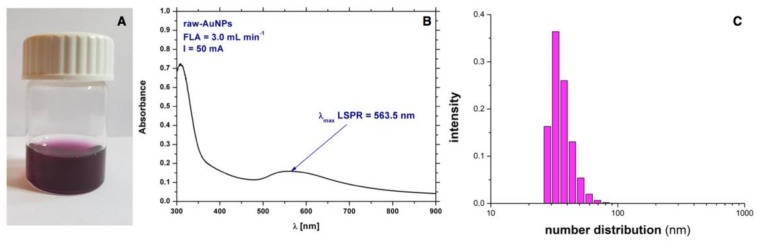
Characteristics of synthesized raw-AuNPs (**A**) visual observation; (**B**) the ultraviolet/visible (UV–Vis) spectrum of Au nanostructures; and (**C**) size distribution by number of raw-AuNPs as determined by DLS.

The initial colour of the 75 mg·L^−1^ solution of Au(III) ions was yellowish. After the impact of dc-APGD, the colour became ruby-red, which is characteristic of solutions containing spherical AuNPs (Figure 2A) [46] These visual observations provided preliminary confirmation of the production of Au nanostructures. 

To examine the optical properties of the solution after dc-APGD treatment, UV–Vis absorption spectroscopy was applied. In Figure 2B, a localized surface plasmon resonance (LSPR) absorption band can be seen, which is characteristic in the range from 520–570 nm for AuNPs [47]. The wavelength of the LSPR absorption band at its maximum (λ_max_) was located at 563.5 nm for the dc-APGD treated solution and its absorbance value was 0.159 (Figure 2B). Based on the profile and intensity of the LSPR absorption band mentioned, it was possible to obtain preliminary information on the size and shape of the synthesized AuNPs. The symmetrical shape of the LSPR absorption band indicated that monodisperse and uniform AuNPs were likely synthesized [15,16]. However, the low absorbance value of the LSPR band at its λ_max_ suggested a rather low efficiency of AuNPs production. To establish the average size of raw-AuNPs as well as the PDI, DLS was used. As can be seen in Figure 2C, the average size by number is determined to be 39.24 ± 8.37 nm. The assessed PDI value was 0.485 ± 0.185, which might indicate the presence of NPs with a non-uniform size distribution. 

The ξ-potential of the raw-AuNPs synthesized *via* dc-APGD was assessed in order to reveal their surface charge. The ξ-potential of the raw-AuNPs was −9.76 ± 1.24 mV, indicating a negative surface charge. The negative ξ-potential value is associated with the mechanism of dc-APGD-mediated AuNP synthesis, as was previously described by Dzimitrowicz et al. [17]. Briefly, the reduction of Au(III) ions to metallic Au(0) of nanometric size is caused by the electrons (e^−^_gas_) injected from the dc-APGD onto the surface of the FLA solution, which contained the AuNPs precursor. The mentioned e^−^_gas_ would have then been thermalized and stabilized as aqueous electrons (e^−^_aq_) due to electron-dipole interactions [10,11]. These electrons are expected to have then interacted with the surface of the AuNPs, resulting in their negative surface charge. 

### 3.2. Fourier Transform Infrared Spectroscopy (FT-IR) Analysis of Obtained Materials

**Figure 3:**
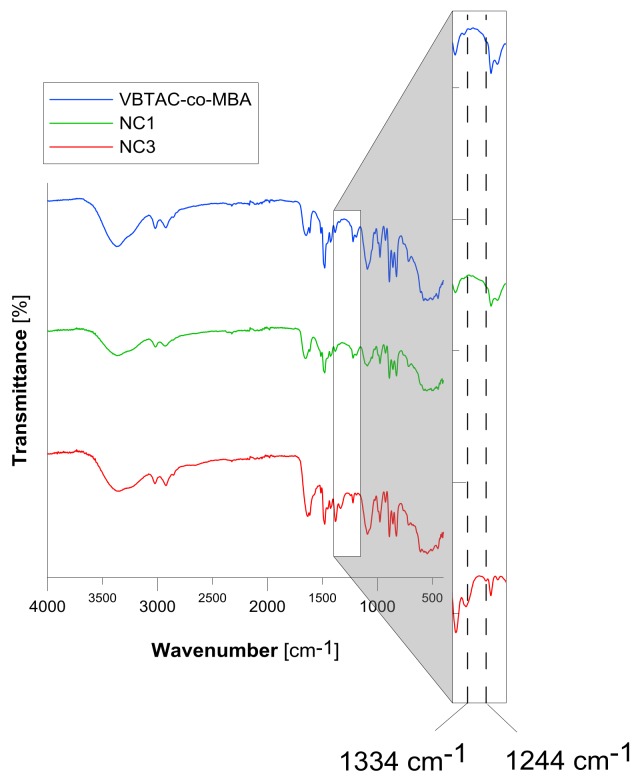
Fourier Transform Infrared Spectroscopy (FT-IR) spectra recorded for raw the VBTAC-*co*-MBA copolymer and the Au/VBTAC-*co*-MBA nanocomposites NC1 and NC3.

To determine the chemical structures of the synthesized hydrogels, FT-IR spectra in KBr pellets were recorded. Figure 3 displays the respective spectra of the VBTAC-co-MBA and Au/VBTAC-*co*-MBA (NC1 and NC3) samples recorded in the range of 4000–400 cm^−1^, respectively. Additionally, in order to prove presence of AuCl_4_^−^ ions, the spectra in the range of 600–100 cm^−1^ were also recorded (data not shown). The positioning of bands relevant for the present study is summarized in Table 1. All polymers revealed wide bands in the area of 3000–3500 cm^−1^ (Figure 3) attributed to O–H and N–H stretching vibrations. Characteristic peaks located at 1479 and 997 cm^−1^ confirmed the presence of –N^+^(CH_3_)_3_ moieties and the aromatic ring, respectively, derived from VBTAC. The bands in the range of 1651–1633 cm^−1^ were attributed to amide (C=O; N–H) groups derived from the cross-linking reagent (MBA). As recorded FT-IR patterns indicated the presence of specific functionalities derived from both monomers used, it was concluded that synthesis of the VBTAC-*co*-MBA resin was successful. It must also be mentioned that all the samples revealed sharp peaks at 1090 cm^−1^ [48]. This was attributed probably to silicone/silica contamination introduced into samples during their preparation for FT-IR analysis, by scratching of the glass petri dish by the dry polymers.

Despite extensive washing after the synthesis of samples NC1 and NC3, the presence of the AuCl_4_^−^ ions (based on multiple bands in the range of 580–345 cm^−1^, absent in spectra recorded for raw VBTAC-*co*-MBA) was noted. This indicated that the unreacted precursor had to participate in the ion exchange process; thus, after synthesis, it could not simply be washed away. Because of the hydrochloride form of VBTAC, the aforementioned ion-exchange reaction was expected to result in creation of HCl according to the following equation [29,30,32,49]:

R-N^+^(CH_3_)_3_ Cl^−^ + HAuCl_4_ = R-N^+^(CH_3_)_3_AuCl_4_^−^ + HCl (2)

This phenomenon was very unfortunate, as excessive HCl might digest AuNPs [50], preventing the formation of nanocomposite. To overcome this issue, polymerization-driven immobilization of synthesized AuNPs was modified by carrying out the process until the gelation point (the sample NC2 was obtained) or by adding NaOH to the reaction mixture (and the sample NC3 was obtained). The spectrum recorded for the sample NC2 revealed no relevant difference compared to the pattern achieved in the case of the sample NC1; therefore, it was not shown here. However, as can be seen in Figure 3 and Table 1, the spectrum recorded for the polymer NC3 revealed a characteristic band attributed to vibrational deformations of the hydroxide group (1334 cm^−1^). Also, the second band indicating the presence of –OH deformations (1244 cm^−1^) can be noticed; however, this may indicate interactions of hydroxyl ions with VBTAC functionalities attached to the benzene ring [48]. Hence, it was concluded that NaOH introduced into the reaction mixture was convenient for the process because it participated in the AuNPs immobilization mechanism. 

### 3.3. Transmission Electron Microscopy (TEM) Photomicrographs of the Obtained Materials 

**Figure 4:**
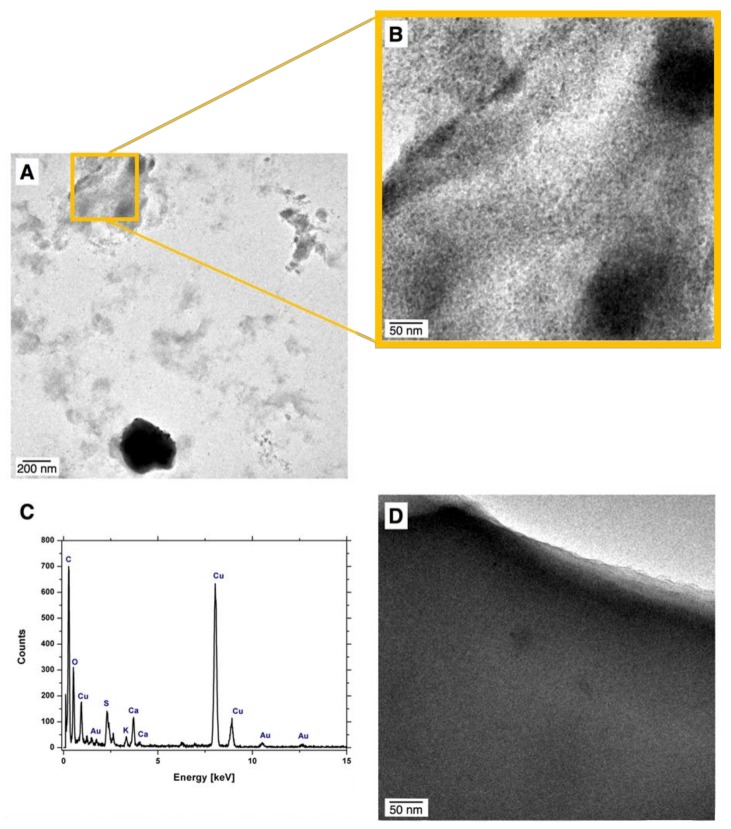
The characteristics of the produced nanocomposites; (**A**,**B**) transmission electron microscopy (TEM) photomicrographs of the NC3 sample; (**C**) energy dispersive X-ray (EDS) analysis of the region presented in Figure 4B; and (**D**) TEM photomicrograph for the NC1 sample.

Figure 4 displays TEM photomicrographs for samples NC1 and NC3, as well as results of EDS analysis of the sample NC3. No AuNPs were observed in the sample NC1, hence the synthesis procedure (i) was not successful at all (Figure 4D). TEM images revealed the presence of raw-AuNPs dispersed within the hydrogel matrix of the sample NC3 (Figure 4A,B). It was evaluated that the average size of raw-AuNPs synthesized *via* dc-APGD and immersed into quaternary hydrogel was 9.31 ± 16.05 nm. The produced Au nanostructures were approximately spherical (95%) in shape (Figure 4A,B). In order to determinate the composition of the sample NC3, the EDS was applied. The presence of peaks corresponding to Au further confirmed that AuNPs were embedded within hydrogel of the sample NC3 (Figure 4C). Additionally, peaks corresponding to O, C, Cu, S, Ca and K were found. The presence of Cu related to the Cu grid onto which the sample was placed. The remaining elements originated from monomers and the free-radical initiator. Slight differences between the results of sizes assessed by using DLS and TEM were noted. It was observed that the average size of raw-AuNPs determined with DLS was larger (≈39 nm) than that determined with TEM. A similar discrepancy in size of Au nanostructures measured with both techniques was already reported in literature [51]. According to the DLS principles, light is scattered on analyzed NPs and therefore reflects the combined size of the raw-AuNPs. In contrast, the TEM measurements are solely based on the size of the metallic Au nanostructures and, therefore, better reflect the actual size of the produced AuNPs.

Based on differences in the structures of functionalities revealed during FT-IR analyses, as well as the fact that the sample NC1 did not contain any Au nanostructures (Figure 4D) while synthesis of the sample NC3 indeed resulted in obtaining nanocomposite, it was possible to determine the mechanism of synthesis routes.

### 3.4. Mechanism of Synthesis of Polymeric Nanocomposite 

During the current work, three different approaches for the synthesis of hydrogel-based nanocomposites containing raw-AuNPs were applied. Although all polymers were prepared by using the AuNPs solution as a reaction environment for polymerization, not all methods were successful in producing Au/VBTAAC-*co*-MBA polymer (Figure 4). Based on differences in the outcomes achieved, it was possible to propose the mechanisms of the synthesis. 

**Figure 5:**
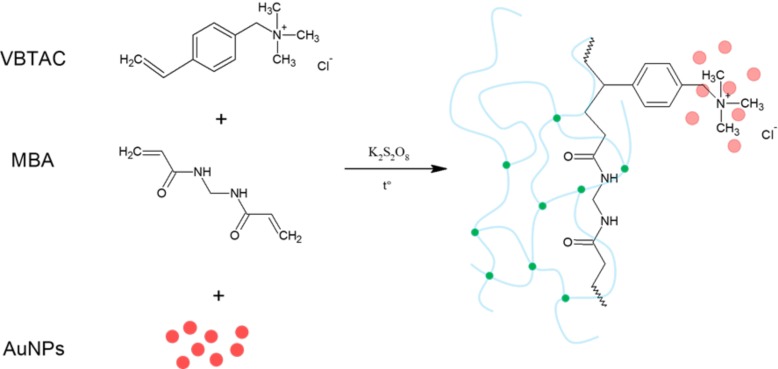
The synthesis route of VBTAC-*co*-MBA nanocomposite that could occur if the reaction environment contained only AuNPs dispersed in water.

Figure 5 displays the expected VBTAC-*co*-MBA synthesis route if the reaction environment contained only Au nanostructures dispersed in water.

**Figure 6:**
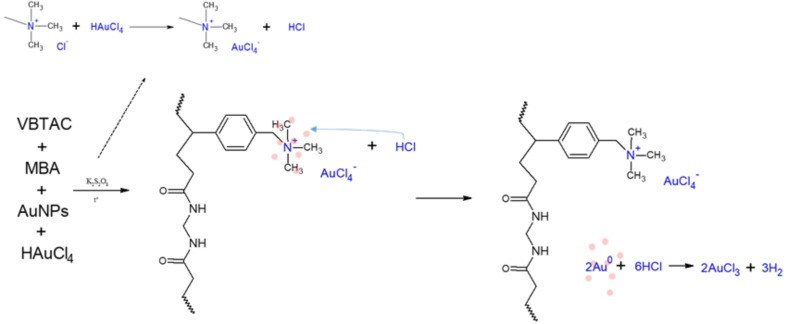
Reactions occurring during synthesis of the sample NC1 (24 h synthesis process).

The free radical copolymerization of VBTAC and MBA likely resulted in formation of a 3-dimensional crosslinked polymeric network that was insoluble in water. As the AuNPs introduced together with the reaction environment posses a negative charge, they were attracted by electrostatic interactions to quaternary ammonium groups [28]. These functional groups played a stabilizing role, holding NPs within the created polymeric network in their unchanged form. However, as revealed on TEM photomicrographs, the sample NC1, synthesized using procedure (i), contained no AuNPs, whereas the sample NC3 successfully stabilized metallic structures. The reason for this difference is that the hydrochloride form of VBTAC was used, and because the reaction environment also contained the unreacted precursor in its dissociated form, i.e., AuCl_4_^−^. Based on TEM photomicrographs and the monitoring of the pH of the reaction mixture, the reactions that occurred during synthesis of the sample NC1, which were not as those shown in Figure 5, were proposed and are displayed in Figure 6.

**Figure 7:**
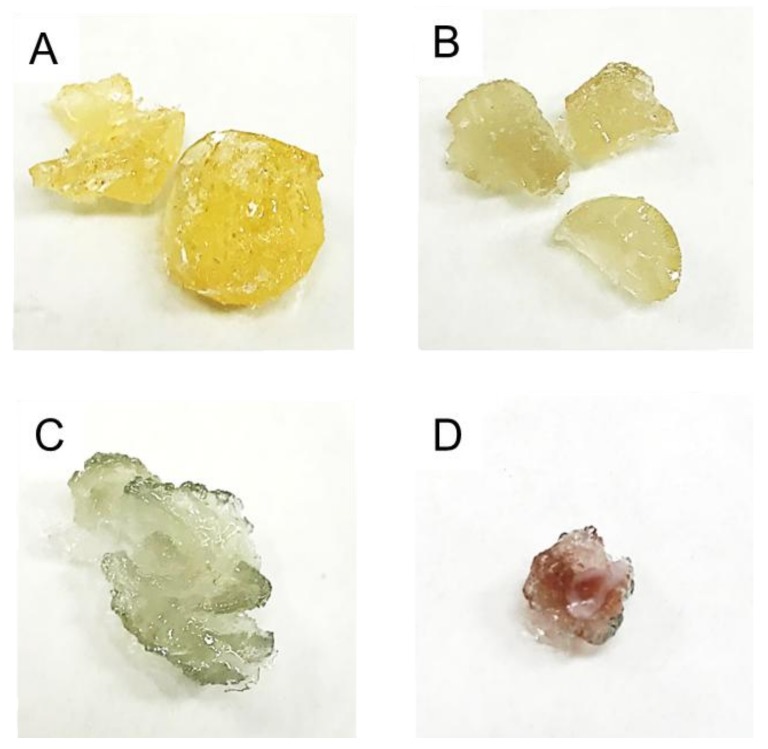
Resultant dried products received after applying routes for synthesis of (**A**) raw VBTAC-*co*-MBA copolymer as well as the samples (**B**) NC1; (**C**) NC2; and (**D**) NC3, respectively.

After introducing the solution of AuNPs and AuCl_4_^−^ into the monomers, VBTAC strong base functionalities exchange anionic tetrachloroaureate(III). This resulted in the formation of HCl (based on Equation (2)). Ion-exchange reactions occurred until equilibrium was reached. After this, the remaining excessive HCl started to digest the AuNPs back into AuCl_4_^−^ ions [50]. As a result, the pH of the reaction mixture dropped dramatically, and synthesized hydrogel contained Au only in its anionic form; thus, no nanocomposite was produced. Consistent with this mechanism, the initial ruby-red colour (associated with the presence of AuNPs) of the sample NC1 disappeared throughout the polymerization process. This resulted in a yellowish polymer, as displayed in Figure 7A, and no metallic species were observed on TEM photomicrographs (see Figure 4D). Hence, it could be stated that the route taken for the synthesis of the sample NC1 was not sufficient for the synthesis of the nanocomposites. 

To overcome this challenge, the second reaction procedure (ii) approach was applied. All reagents were held together in the reactor for a short time, long enough just to allow monomers to reach the gelation point (approximately 30 min). After this, the hydrogel formed was extracted from the flask and washed extensively with water to remove excessive HCl preventing digestion of AuNPs. The resultant product was initially characterized by an intensive ruby-red colour that originated from the AuNPs. Thus, extensive washing of the sample NC2 was not sufficient to prevent the ion-exchange reactions because the polymer eventually lost its ruby-red colour and appeared as a greyish-like solid (Figure 7C). Moreover, the short polymerization time raised doubts about the average molecular weight of this polymer, and thus its applicability. Based on this it could be stated that the approach (ii) only decreased the rate of ion-exchange reactions, and thus the generation of HCl.

**Figure 8:**
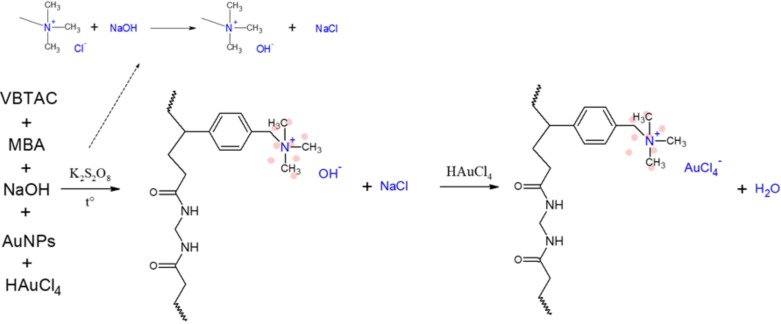
Reactions occurring during synthesis of the sample NC3 (addition of NaOH).

As revealed above, synthesis of the nanocomposite must account for ion-exchange reactions occurring within the hydrogels created. Therefore, the approach (iii) was aimed to force these reactions to take another route, as displayed in Figure 8. 

Based on FT-IR spectra (Figure 3), the addition of NaOH forced ion-exchange reactions leading to the substitution of OH^−^ to amine located at the aromatic ring, which resulted in the formation of NaCl. It could not be excluded that amines first exchanged AuCl_4_^−^; however, due to the ionic strength of these ions, functionalities should prefer NaOH instead of tetrachloro aureate(III) [49]. Consistent with this, the FT-IR spectra recorded for the sample NC3 (Figure 3) did reveal additional deforming vibrations of hydroxides originating from NaOH; thus, the potential influence of HCl (if formed) was negligible. Nevertheless, because the reaction mixture still contained the unreacted precursor, excess NaOH was introduced into the mixture to prevent the formation of HCl. As time passed, the remaining AuCl_4_^−^ ions must eventually be exchanged due to its strong affinity towards amino groups [29,30,31]. However, because of the earlier reaction, only water is generated. As displayed in Figure 7D, the synthesized hydrogel adopted the ruby-red colour of the solution containing AuNPs and, as can be seen in the TEM micrographs, metallic Au was successfully immobilized within the polymeric matrix in its original form.

### 3.5. Evaluation of the Heat-Transfer Rate 

Hydrogels facilitate heat transfer due to their ability to undergo volume-phase transition between hydrophilic-hydrophobic states [41,52,53] and efficiently transfer radiation energy into heat [54]. These properties of hydrogels offer unique applications in micromechanics and microelectronics, where efficient transferring of heat is particularly important. However, past work indicated that the synthesis of nanocomposites could further enhance the thermal properties of various materials [19,54,55].

**Figure 9:**
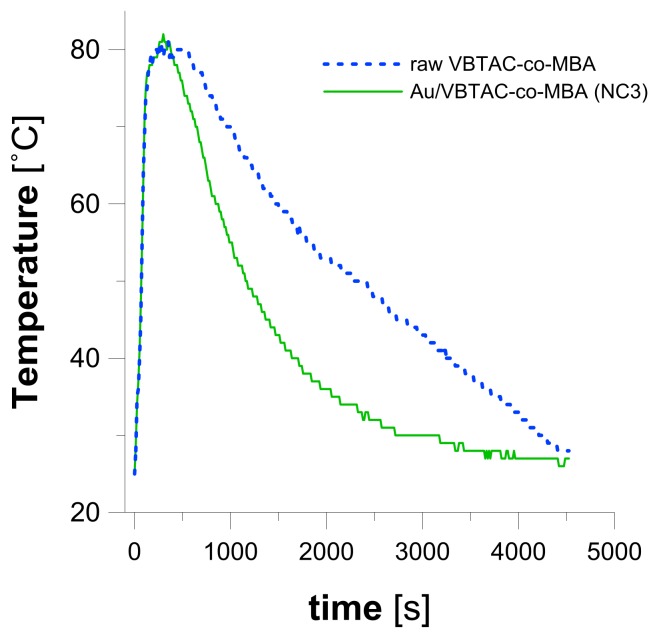
Heating/cooling curves registered for the raw VBTAC-*co*-MBA and Au/VBTAC-*co*-MBA (NC3).

To evaluate the possible use of prepared Au/VBTAC-*co*-MBA nanocomposite in heat-exchange processes, samples of the raw hydrogel and nanocomposite (NC3) were heated in a microwave reactor up to 80 °C, and then cooled down in a stream of air to 40 °C. Figure 9 displays the heating/cooling curves registered for the both tested materials. Experimental data fitted within the range of 80–40 °C (>300 s, Figure 9) were re-calculated using Equation (1) in order to obtain the cooling rate constant (*k*_he_). Based on the outcomes of the analysis, it was established that the raw VBTAC-*co*-MBA copolymer was cooled down at a rate of 1.5 × 10^−3^ s^−1^. When applying the same conditions to nanocomposite NC3, the rate of cooling was increased to 3.3 × 10^−3^ s^−1^. This greater than two-fold difference in cooling rates is reflected by plots displayed in Figure 9, which clearly display that nanocomposite NC3 requires almost half an hour less to reach 40 °C than its equivalent without AuNPs. This effect was attributed to the introduction of NPs and their improvement effect in heat-transfer processes [18,19,20,21]. However, in the present study AuNPs were immobilized within cross-linked hydrogel, and thus the resulted nanocomposite could overcome issues related to the stability and separation of NPs; thus, they might be much easier to operate in real-life conditions than nanofluids. Results obtained within the present study complied with data found in literature, reporting a significant (from 30% to 300%) increase of the heat-transfer rate on polyaniline- [55], poly(*N*-isopropylacrylamide)- [54] and stearic acid-based [19] nanocomposites containing CuO, and graphene nanoparticles, respectively.

## 4. Conclusions

A new method was developed for the immobilization of the raw-AuNPs, synthesized by dc-APGD operated in a continuous-flow reaction-discharge system, into a quaternary ammonium-based hydrogel. The procedure involved free-radical polymerization of VBTAC and MBA monomers in the solution where Au nanostructures were dispersed. Polymerization-driven immobilization faced obstacles of ion-exchange reactions occurring between the strong base functionalities of VBTAC and the unreacted gold precursor. This resulted in the formation of HCl that further digested the AuNPs. 

The successful synthesis method forced the ion-exchange reactions to take another route by introducing NaOH into the reaction mixture. As a result, raw-AuNPs produced *via* dc-APGD were successfully dispersed in the solution of monomers, and then immobilized within the produced resin, providing a new, facile method for the synthesis of hydrogel-based nanocomposites. To the best of our knowledge, the polymerization-driven immobilization of dc-APGD AuNPs described here is being reported for the first time in the literature. 

The synthesized Au/VBTAC-*co*-MBA nanocomposite has potential utility in heat-transfer processes, as its cooling rate was more than twice that of the raw VBTAC-*co*-MBA hydrogel. This makes the resultant nanocomposite potentially applicable in various micro-technologies, where efficient cooling is of great concern.

## 5. Patents

The AuNPs were produced *via* a method protected by the Polish patent application No. P.417933.

## Figures and Tables

**Table 1:** Summarized FT-IR data for synthesized materials.

Functionality	Wavenumber [cm^−1^]		
	VBTAC-*co*-MBA	Au/VBTAC-*co*-MBA	
		NC1	NC3
R-N^+^(CH_3_)_3_	1479	1479	1479
N–H (amide deformation)	1513	1513	1513
C=O (amide stretching)	1648	1651	1633
AuCl_4_^−^	-	580–345 ^#^	
–OH (deformation)	-	-	1334 1244

^#^ Based on spectra recorded in the range of 600–100 cm^−1^. Spectra are available from the corresponding author on request.

## Abbreviations

APP	atmospheric pressure plasma
AuNPs	gold nanoparticles
Au/VBTAC-*co*-MBA	nanocomposite containing gold nanparticles immobilized within (vinylbenzyl)trimethylammonium chloride-*co*-*N*,*N*-methylenebisacrylamide copolymer
dc-APGD	direct current atmospheric pressure glow discharge
DLS	dynamic light scattering
EDS	energy-dispersive X-ray spectroscopy
FLA	flowing liquid anode
FT-IR	Fourier transformation infrared spectroscopy
ID	internal diameter
LSPR	localized surface plasmon resonance
MBA	*N*,*N*-methylenebisacrylamide
NFs	nanofluids
NMs	nanomaterials
NPs	nanoparticles
OD	outside diameter
RONS	reactive oxygen and nitrogen species
TEM	transmission electron microscopy
UV–Vis	ultraviolet-visible
VBTAC	(vinylbenzyl)trimethylammonium chloride
